# Liquid-solid joining of bulk metallic glasses

**DOI:** 10.1038/srep30674

**Published:** 2016-07-29

**Authors:** Yongjiang Huang, Peng Xue, Shu Guo, Yang Wu, Xiang Cheng, Hongbo Fan, Zhiliang Ning, Fuyang Cao, Dawei Xing, Jianfei Sun, Peter K. Liaw

**Affiliations:** 1 State Key Laboratory of Advanced Welding and Joining, Harbin Institute of Technology, China; 2 School of Materials Science and Engineering, Harbin Institute of Technology, China; 3 Key Laboratory of Micro-Systems and Micro-Structures Manufacturing (Harbin Institute of Technology), Ministry of Education, China; 4 Department of Materials Science and Engineering, University of Tennessee, Knoxville, USA

## Abstract

Here, we successfully welded two bulk metallic glass (BMG) materials, Zr_51_Ti_5_Ni_10_Cu_25_Al_9_ and Zr_50.7_Cu_28_Ni_9_Al_12.3_ (at. %), using a liquid-solid joining process. An atomic-scale metallurgical bonding between two BMGs can be achieved. The interface has a transition layer of ~50 μm thick. The liquid-solid joining of BMGs can shed more insights on overcoming their size limitation resulting from their limited glass-forming ability and then promoting their applications in structural components.

Bulk metallic glasses (BMGs), also termed as bulk amorphous alloys, are a kind of advanced materials with a disordered atomic-scale structure^1^,^2^,^3^,^4^. The unique microstructure of BMGs often offers their fantastic properties, like ultrahigh (near theoretical) fracture strength, superior elastic limit, and exceptional corrosion/wear resistance^5^,^6^,^7^,^8^,^9^,^10^. Therefore, BMGs have currently stimulated widespread interests from the materials-science community due to their technological promise for industrial applications and scientific importance in unveiling the glass-formation and glass phenomena^11^,^12^,^13^,^14^,^15^,^16^,^17^,^18^. However, sufficiently-high critical cooling rates (>10^3^ K/s) are typically required to fabricate these glasses from the molten alloys, using a rapid solidification technique to achieve an amorphous microstructure, which seriously restricts the shapes of the obtained samples within ribbons, wires, or powders. The size limitation of BMGs has become a stumbling block for their practical applications^19^. To address this critical issue, over the past decades, tremendous research efforts have been devoted to the joining of BMGs to obtain industrial-size samples through various techniques^20^,^21^,^22^,^23^,^24^,^25^,^26^,^27^,^28^,^29^,^30^,^31^. Until now, the BMGs in several alloy systems have been successfully welded together by a liquid-state process using electron beams^20^, laser beams^21^, and pulse current methods^22^, or by a solid-state process using friction^23^,^24^,^25^, explosion^26^, thermoplastic deforming^27^, and ultrasonic welding methods^28^. For instance, Kawamura *et al*.^29^,^30^ have succeeded in welding Pd_40_Ni_40_P_20_, Pd_40_Cu_30_Ni_10_P_20_, Zr_55_Al_10_Ni_5_Cu_30_ and Zr_41_Ti_14_Cu_12_Ni_10_Be_23_ (atomic percent, at. %) BMGs to the same ones by the friction method, Chen *et al*.^27^ introduced a novel thermoplastic-deforming method to join BMGs, Wang *et al*.^21^ welded 3-mm thick TiZrNiCuBe BMG plates without defects or crystallization by the laser-welding process, Kawamura *et al*.^20^ also welded a 3.5 mm thick Zr_41_Ti_14_Cu_12_Ni_10_Be_23_ BMG plate with an amorphous structure and full strength by electron beam welding, and Zhou *et al*.^22^ connected Zr_55_Al_10_Ni_5_Cu_30_ BMG samples using the pulse-current method. Special attempts should be focused on the welding/joining techniques of two different materials, such as joining a BMG sample to another one with different chemical compositions, to achieve the excellent combination of specific properties of the individual material of the welded parts and then to provide flexible design possibilities for high-performance products.

Here, we introduce a new liquid-solid joining method in which we directly cast a glass-forming melt into a copper mould inserted by a solid-state glassy rod with another chemical composition. At the moment of solidification, the outer surface of the solid-state glassy rod involves an instantaneous liquid-solid interface reaction. After joining, the microstructures and mechanical properties of the welded samples were investigated. The interface characterization between two BMGs was analyzed. The mechanism for the metallurgical bonding upon the liquid-solid joining method was discussed in details. It is expected that the method presented here could provide more freedom in finely-tailoring the microstructures and mechanical properties of BMG products.

## Methods

**Figure 1:**
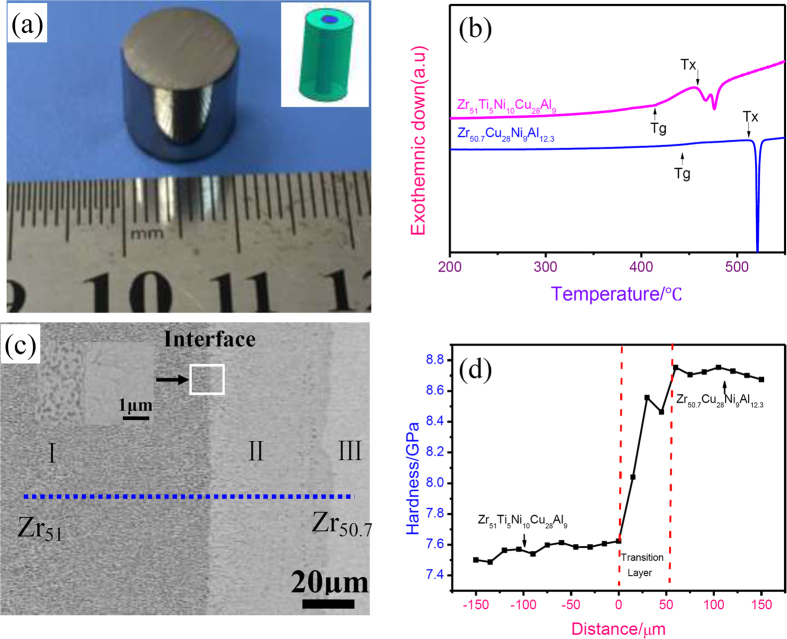
(**a**) Outer appearance of the composite sample with the outer Zr_50.7_ alloy and the inner Zr_51_ alloy fabricated using a liquid-solid joining process with the inset illustrating the dimension of the joint, (**b**) DSC curves obtained from the inner Zr_51_ and outer Zr_50.7_ alloy parts of the welded sample at a constant heating rate of 20 °C/min, (**c**) SEM image obtained from the cross section of the Zr_51_/Zr_50.7_ alloy joint with the inset showing the high magnification SEM of the interface, and (**d**) Nano-hardness profile across the interface of the welded Zr_51_/Zr_50.7_ alloy joint (the position, *x* = 0 corresponds to the interface).

The alloy ingots of the nominal compositions, Zr_51_Ti_5_Ni_10_Cu_25_Al_9_ and Zr_50.7_Cu_28_Ni_9_Al_12.3_ (at. %, hereafter denoted as Zr_51_ and Zr_50.7_, respectively) were obtained by arc-melting a mixture of Zr, Ti, Ni, Cu, and Al with high purity (>99.5%) in a Ti-gettered argon atmosphere. Each ingot was re-melted and electromagnetically stirred at least four times to ensure the chemical homogeneity. Firstly, the Zr_51_ alloy cylinders of 4 mm in diameter and ~30 mm in length were fabricated by drop-casting the molten alloys into a copper mould. Then, the obtained cast Zr_51_ alloy cylinder was inserted into another copper mould with a hole of 10 mm in diameter. The Zr_50.7_ alloy melt was cast into this mould, to fabricate a composite sample with the outer Zr_50.7_ alloy of 10 mm in diameter and the inner Zr_51_ alloy of 4 mm in diameter, as presented in Fig. 1a. The thermal properties of the Zr_51_ and Zr_50.7_ alloy parts of the composite sample were examined using a Perkin-Elmer differential scanning calorimetry (DSC) at a heating rate of 20 K/min under a flow of purified argon atmosphere. The joining area of the composite sample was examined by scanning electron microscopy (SEM, FEI Sirion) equipped with the energy-dispersive spectrometry (EDS, Oxford INCA). To study the structure and phase nature of the post-welded composite material, two samples containing the welding interface were sliced by the dual beams focused ion beam (FIB) system (FEI HELIOS NanoLab 600i) and then subjected to a transmission electron microscopy (TEM, JOEL JEM-2100) observations at 200 keV.

In order to characterize the mechanical properties of the welded joint, room-temperature nanoindentation tests were performed on its polished cross-section at a constant loading rate of 0.5 mN/s with a maximum applied load of 10 mN using an MTS Nano indenter XP system with a Berkovich diamond indenter. The loading and unloading rates were kept the same, and a holding time of 5 s was used at the maximum load. The nano-hardness and elastic modulus were calculated, using the Oliver-Pharr method^32^.

## Results

Figure 1b shows the DSC curves obtained from the Zr_51_ and Zr_50.7_ alloy parts of the composite sample at a heating rate of 20 K/min. A distinct glass transition followed by a wide supercooled liquid region and exothermic events due to crystallization events can be observed in the thermograms for the two alloys. Glass transition temperature (*T*_g_) and crystallization temperature (*T*_x_) are determined to be 407 °C and 450 °C for the Zr_51_ alloy, and 443 °C and 501 °C for the Zr_50.7_ alloy, respectively.

Figure 1c is the SEM image of the cross section of the welded sample. No any defects, voids, or cracks can be detected at or near the interface, revealing that two BMG materials exhibit a perfect and strong metallurgical bonding using the liquid-solid joining method. It can be contributed to the fact that the liquidus temperature, *T*_l_, of the Zr_50.7_ alloy (841 °C) is 110 °C higher than that of the Zr_51_ alloy (731 °C). When the Zr_50.7_ alloy melts was drop cast into a copper mould inserted by the solid-state Zr_51_ alloy, a transient melting process will inevitably take place on the surface area of the Zr_51_ alloy cylinder, favoring the mutual diffusion of the atoms of the two alloys. Clearly, the microstructure of the welded sample can be divided into three different regions I, II, and III. In region I, nanocrystals with an average size of ~200 nm are embedded in the featureless glassy matrix, as shown in Fig. 1c inset. Region II with a width of ~50 μm exhibits a columnar crystal structure, whereas region III has a typical featureless nature of the amorphous phase. The nano-mechanical properties of the welded joint were characterized using nanoindentation. Figure 1d presents the nano-hardness profile across the interface of the Zr_51_/Zr_50.7_ alloy joint, where the origin point of the *x*-axis denotes the interface of the Zr_51_/Zr_50.7_ alloys. As seen, the nano-hardness values of the regions in Zr_51_ and Zr_50.7_ alloy parts far away from the interface are 7.4 GPa, and 8.7 GPa, respectively. The region adjacent to the interface with a width of ~50 μm has a nano-hardness value ranging from 7.4 to 8.7 GPa, further confirming the width of transition layer shown in Fig. 1c.

**Figure 2:**
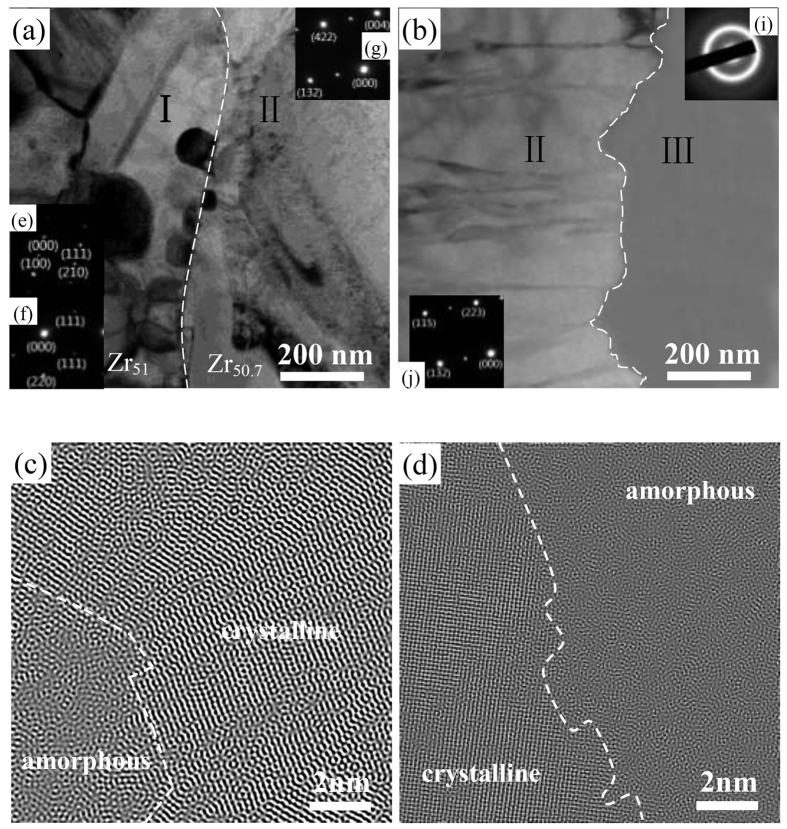
TEM images: The bright-field images of the interface between (**a**) region I and region II, and (**b**) region II and region III, (**c,d**) the corresponding Fast Fourier Transformation filtered high-resolution TEM image, with the white dashed lines showing the boundary between regions I, II, and III, (**e,f**) the SAED patterns taken from the crystals in region I, (**g,h**) the SAED patterns taken from the columnar crystals in region II, and (i) the SAED pattern taken from region III.

To study the structure and phase nature of the post-welded material, two samples containing the interface of regions I and II, and region II and III, respectively, were sliced by the FIB and then subjected to TEM observations. Figure 2a,b show the bright-field images of the interface between regions I and II, and region II and III, respectively. Figure 2c,d are the corresponding Fast Fourier Transformation filtered high resolution TEM image. The white dashed lines drawn in Fig. 2a–d show the clear boundary between region I and region II, and region II and region III, respectively. It can be clearly seen that there is no any traces of voids and cracks at the interface, which demonstrates perfectly metallurgical bonding. For region I, many nano-crystals can be found to be embedded in the glassy matrix [See Fig. 2a,c]. Columnar crystals are found in region II [See Fig. 2b]. Figure 2e,f are the SAED patterns taken from the crystals in region I. The strong diffraction spots can be identified as the Al_4_Cu_9_ phase (PDF: 65-3347) with lattice parameters of *a* = 8.704 nm, *b* = 8.704 nm, and *c* = 8.704 nm and the Al_3_Zr_2_ phase (PDF: 65-1431) with lattice parameters of *a* = 9.601 nm, *b* = 13.906 nm, and *c* = 5.574 nm. Figure 2g,h are the SAED patterns taken from the crystals in region II close to the interface between region I and region II, and region II and region III, respectively. The diffraction spot of crystals in Fig. 2g,h can be identified to be the same phase, Cu_10_Zr_7_ (PDF: 47-1028), with lattice parameters of *a* = 12.675 nm, *b* = 9.313 nm, and *c* = 9.347 nm. Figure 2i is the SAED pattern of region III, showing clearly diffuse halo rings, typical of an amorphous structure. It suggests that region III maintains its amorphous structure without any devitrification after the liquid-solid joining process.

**Figure 3:**
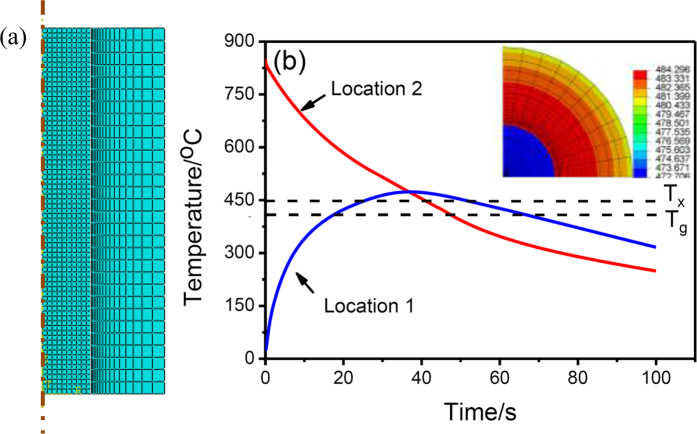
(**a**) 2D-square mesh used in the temperature-field modeling, and (**b**) the temperature profiles extracted from two locations: the center of the inner Zr_51_ alloy (Location 1), and another one locates in the outer Zr_50.7_ alloy, which is 50 μm away from the Zr_51_/Zr_50.7_ interface (Location 2), with inset showing the temperature distribution of the Zr_51_ and Zr_50.7_ alloys after 30 s drop-casting the Zr_50.7_ alloy melt into the copper mould.

The above experimental results indicate that the Zr_51_ and Zr_50.7_ alloys can be metallurgically bonded at the atomic scale by the liquid-solid joining process. In order to make clear the bonding mechanism of two different BMG samples, numerical simulations of the joining process have also been performed, using the Abaqus software based on the following Fourier’s heat conduction equation^33^,


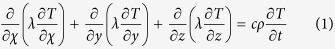


where *ρ*, *c*, and *λ* are the density, the specific heat, and the thermal conductivity, respectively. For such a liquid-solid joining process, the Zr_50.7_ alloy melt can be considered as the heat source of the whole system. During the joining process, the heat of the Zr_50.7_ alloy melt conducted to the solid-state Zr_51_ alloy and Cu mold. The initial temperature of the Zr_50.7_ alloy melt, solid-state Zr_51_ alloy and copper mold are assumed to be 850 °C, 25 °C, and 25 °C, respectively. Figure 3a shows the two-dimensional (2D) square meshed used in the temperature field analysis and much smaller meshes with the minimum mesh size of 5 μm were used in the area near the interface to study the thermal history during the joining process. Figure 3b shows the temperature profiles extracted from two locations. One is the center of Zr_51_ alloy (Location 1), and the other locates in the Zr_50.7_ alloy where is 50 μm away from the Zr_51_/Zr_50.7_ interface (Location 2). Figure 3b inset presents the temperature field within the Zr_50.7_ alloy melt, and the solid-state Zr_51_ alloy at 30 s after the Zr_50.7_ alloy melt is drop cast into the copper mould.

## Discussion

During the liquid-solid joining process, the highest temperature of the Zr_51_ alloy is 473 °C, higher than *T*_x_ of the Zr_51_ alloy. It facilitates the crystallization event during the initial joining stage. Thus, a structure of numerous crystals embedded in the glassy matrix can be detected in the Zr_51_ alloy after joining, as seen in Figs 1c and 2a.

Next, we will discuss the structure evolution in outer liquid-state Zr_50.7_ alloy during joining. For Location 2, the time duration from 850 °C down to 446 °C, *T*_g_ of the Zr_50.7_ alloy, is 41 s, yielding a cooling rate of 9.85 °C/s, almost identical with its critical cooling rate for glass formation (9.8 °C/s)^34^. Whereas, for the areas far away from the interface, a high cooling rate (>9.8 °C/s), due to the severe heat conduction between the Zr_50.7_ melt and the copper mould, ensures the Zr_50.7_ alloy to preserve its amorphous structure after joining (See Region III in Fig. 1c). The temperature of the inner solid-state Zr_51_ alloy quickly increases from 25 °C to 473 °C. After that, the temperature in the Zr_51_ alloy is even higher than that in the Zr_50.7_ alloy, as seen in Fig. 3b. The heat will be continuously conducted from the Zr_51_ alloy to the Zr_50.7_ alloy until the end of the joining process. Thus, the locations adjacent to the interface, especially those with a distance of <50 μm to the interface, will experience a lower cooling rate than those away from the interface, resulting in the formation of crystalline phases after joining (See Region II in Fig. 1c). Meanwhile, due to the existence of a great temperature gradient and, thus, a large concentration gradient from the interface to the copper mould, the growth direction of the crystalline phase should be perpendicular to the interface. Therefore, the columnar crystals precipitate in the locations adjacent to the interface during joining, as seen in the inset of Figs 1c and 2a,b.

Here, using the proposed liquid-solid joining process, one can easily join two different BMG samples together. The joint sample exhibits an excellent metallurgical bonding, as confirmed in Figs 1c and 2. Based on this process, one could obtain BMG components of desirable properties by fine tuning the chemical composition, size, and shape of the two BMGs. For instance, if one can join Gd-based BMG of excellent functional properties but disappointed mechanical properties^35^ with Co-based BMG of high fracture strength exceeding 6000 MPa^36^ together, to get a composite component with the outer Co-based BMG and the inner Gd-based BMG, the obtained composites would be expected to have both high strength and high magnetocaloric effect. On the other hand, the liquid-solid joining method involves universal arc melting furnace. Despite the fact that the crystallization around the Zr_50.7_/Zr_51_ interface is hardly avoided due to low cooling rate in current conditions, the mechanical properties of the joint did not deteriorate, which is confirmed by the nanoindentation results (See Fig. 1d). In some cases, the plasticity of BMGs can be enhanced by the appropriate control of the size and distribution of crystals. For example, isolated crystallite islands can be created to optimize the mechanical performance of BMGs via surface crystallization induced by surface mechanical attrition treatment process^37^. Improved tensile plastic strain can be obtained by the formation of microstructural heterogeneity induced by cold rolling^38^. The ductility of a Ti-based BMG under compression was found to increase up to ∼42% without reducing the maximum strength due to the existence of nanocrystals induced by isothermal annealing treatment^39^. Based on the tunable chemical composition and corresponding properties, the flexible shape of the joint component, and the simple facility required, although the occurrence of crystallization around the interface, the liquid-solid joining process proposed in the present study are still considered as a useful and powerful method, and can be used to join two dissimilar advanced materials together, such as TiAl intermetallics, high entropy alloys, BMGs, and high temperature Ti alloys.

## Summary

In summary, two typical Zr-based BMGs, Zr_51_Ti_5_Ni_10_Cu_25_Al_9_ and Zr_50.7_Cu_28_Ni_9_Al_12.3_, were successfully joined, using a liquid-solid joining process. There is a gradual change in the microstructure and nanohardness of ~50 μm in thickness across the interface of the joint. The microstructural feature of the joint is interpreted in details, based on the solidification theory and numerical simulation. The present joining process is promising for fabricating composite materials of two different BMGs, with flexible properties.

## Additional Information

**How to cite this article**: Huang, Y. *et al*. Liquid-solid joining of bulk metallic glasses. *Sci. Rep*. **6**, 30674; doi: 10.1038/srep30674 (2016).

## References

[b1] Greer A. L. Metallic glasses. Science 267, 1947–1953 (1995). 17770105 10.1126/science.267.5206.1947

[b2] Wang W. H., Dong C. & Shek C. H. Bulk metallic glasses. Mater. Sci. Eng. R 44, 45–89 (2004).

[b3] Inoue A., Kong F. L., Zhu S. L., Shalaan E. & Al-Marzouki F. M. Production methods and properties of engineering glassy alloys and composites. Intermetallics. 58, 20–30 (2015).

[b4] Kim S.-Y. . Imprinting bulk amorphous alloy at room temperature. Sci. Rep. 5, 16540 (2015). 26563908 10.1038/srep16540 PMC4643295

[b5] Chen C. Q., Pei Y. T. & De Hosson J. Th. M. Apparently homogeneous but intrinsically intermittent flow of taper-free metallic glass nanopillars. Scr. Mater. 67, 947–950 (2012).

[b6] Kuzmin O. V., Pei Y. T. & De Hosson J. T. M. Size effects and ductility of Al-based metallic glass. Scr. Mater. 67, 344–347 (2012).

[b7] Huang Y. J., Khong J. C., Connolley T. & Mi J. The onset of plasticity of a Zr-based bulk metallic glass. Inter. J. Plasticity. 60, 87–100 (2014).

[b8] Huang Y. J., Sun Y. & Shen J. Tuning the mechanical performance of a Ti-based bulk metallic glass by pre-deformation. Intermetallics. 18, 2044–2050 (2010).

[b9] Luo J., Keblinski P. & Shi Y. F. A model metallic glass exhibits size-independent tensile ductility. Acta Mater. 103, 587–594 (2016).

[b10] Yang Y. . Probing stochastic nano-scale inelastic events in stressed amorphous metal. Sci. Rep. 4, 6699 (2014). 25331932 10.1038/srep06699 PMC4204032

[b11] Huang Y. J., Chiu Y. L., Shen J., Chen J. J. J. & Sun J. F. Indentation Creep of a Ti-based Metallic Glass. J. Mater. Res. 24, 993–997 (2009).

[b12] Chen C. Q., Pei Y. T. & De Hosson J. T. M. Effects of size on the mechanical response of metallic glasses investigated through *in situ* TEM bending and compression experiments. Acta Mater. 58, 189–200 (2010).

[b13] Lu Z., Jiao W., Wang W. H. & Bai H. Y. Flow Unit perspective on room temperature homogeneous plastic deformation in metallic glasses. Phys. Rev. Lett. 113, 045501 (2014). 25105632 10.1103/PhysRevLett.113.045501

[b14] Na J. H., Demetriou M. D., Floyd M., Hoff A., Garrett G. R. & Johnson W. L. Compositional landscape for glass formation in metal alloys. PNAS. 111, 9031–9036 (2014). 24927600 10.1073/pnas.1407780111 PMC4078826

[b15] Wang Y. W., Li M. & Xu J. W. Toughen and harden metallic glass through designing statistical heterogeneity. Scr. Mater. 113, 10–13 (2016).

[b16] Wang Y. J., Zhang M., Liu L., Ogata S. & Dai L. H. Universal enthalpy-entropy compensation rule in the deformation of metallic glasses. Phys. Rev. B. 92, 174118 (2015).

[b17] Yu H. B., Richert R., Maaß R. & Samwer K. Unified Criterion for Temperature-Induced and Strain-Driven Glass Transitions in Metallic Glass. Phys. Rev. Lett. 115, 135701 (2015). 26451567 10.1103/PhysRevLett.115.135701

[b18] Huang Y. J. . Structure and mechanical property modification of a Ti-based metallic glass by ion irradiation. Scr. Mater. 103, 41–44 (2015).

[b19] Jiang M. Q., Huang B. M., Jiang Z. J., Lu C. & Dai L. H. Joining of bulk metallic glass to brass by thick-walled cylinder explosion. Scr. Mater. 97, 17–20 (2015).

[b20] Kagao S., Kawamura Y. & Ohno Y. Electron-beam welding of Zr-based bulk metallic glasses. Mater. Sci. Eng. A375–377, 312–316 (2004).

[b21] Wang G., Huang Y. J., Shagiev M. & Shen J. Laser welding of Ti_40_Zr_25_Ni_3_Cu_12_Be_20_ bulk metallic glass. Mater. Sci. Eng. A541, 33–37 (2012).

[b22] Zhou Y. Z., Zhang Q. S., He G. H. & Guo J. D. Connection of bulk amorphous alloy Zr_55_Al_10_Ni_5_Cu_30_ by high current density electropulsing. Mater. Lett. 57, 2208–2211 (2003).

[b23] Shin H.-S. & Jung Y.-C. Characteristics of friction stir spot welding of Zr-based bulk metallic glass sheets. J. Alloys Compd. 504, S279–S282 (2010).

[b24] Wang G., Huang Y. J., Makhanlall D. & Shen J. Friction joining of Ti_40_Zr_25_Ni_3_Cu_12_Be_20_ bulk metallic glass. J. Mater. Proc. Technol. 212, 1850–1855 (2012).

[b25] Wong C. H. & Shek C. H. Friction welding of Zr_41_Ti_14_Cu_12.5_Ni_10_Be_22.5_ bulk metallic glass. Scr. Mater. 49, 393–397 (2003).

[b26] Kawamura Y., Ohno Y. & Chiba A. Development of welding technologies in bulk metallic glasses. Mater. Sci. Forum. 386–388, 553–558 (2002).

[b27] Chen W., Liu Z. & Schroers J. Joining of bulk metallic glasses in air. Acta Mater. 62, 49–57 (2014).

[b28] Kim J. H. Weldability of Cu_54_Zr_22_Ti_18_Ni_6_ bulk metallic glass by ultrasonic welding processing. Mater. Lett. 130, 160–163 (2014).

[b29] Kawamura Y., Shoji T. & Ohno Y. Welding technologies of bulk metallic glasses. J. Non-Cryst. Solids 317, 152–157 (2003).

[b30] Kawamura Y. & Ohno Y. Superplastic bonding of bulk metallic glasses using friction. Scr. Mater. 45, 279–285 (2001).

[b31] Kuo P. H. . Bulk-metallic glasses joining in a supercooled-liquid region. Mater. Chem. Phys. 120, 532–536 (2010).

[b32] Oliver W. C. & Pharr G. M. An Improved Technique for Determining Hardness and Elastic Modulus Using Load and Displacement Sensing Indentation. J. Mater. Res. 7, 1564–1583 (1992).

[b33] Narasimhan T. N. Fourier’s heat conduction equation: History, influence, and connections. J. Earth System Sci. 108, 117–148 (1999).

[b34] Sun Y. J. PhD Thesis of Harbin Institute of Technology, China, 2009.

[b35] Xia L., Guan Q., Ding D., Tang M. B. & Dong Y. D. Magneto-caloric response of the Gd_60_Co_25_Al_15_ metallic glasses. Appl. Phys. Lett. 105, 192402 (2014).

[b36] Wang J. F., Li R., Hua N. B. & Zhang T. Co-based ternary bulk metallic glasses with ultrahigh strength and plasticity. J. Mater. Res. 26, 2072–2079 (2011).

[b37] Fan J. T., Chen A. Y., Wang J., Shen J. & Lu J. Improved plasticity and fracture toughness in metallic glasses via surface crystallization. Intermetallics. 19 1420–1427 (2011).

[b38] Park J. M. . Internal structural evolution and enhanced tensile plasticity of Ti-based bulk metallic glass and composite via cold rolling. J. Alloys Compd. 615, S113–S117 (2014).

[b39] Jun H.-J., Lee K. S., Kim C. P. & Chang Y. W. Ductility enhancement of a Ti-based bulk metallic glass through annealing treatment below the glass transition temperature. Intermetallics. 20, 47–54 (2012).

